# Impact of Deep Coalescence on the Reliability of Species Tree Inference from Different Types of DNA Markers in Mammals

**DOI:** 10.1371/journal.pone.0030239

**Published:** 2012-01-19

**Authors:** Alejandro Sánchez-Gracia, Jose Castresana

**Affiliations:** Institute of Evolutionary Biology (CSIC-UPF), Barcelona, Spain; University of York, United Kingdom

## Abstract

An important challenge for phylogenetic studies of closely related species is the existence of deep coalescence and gene tree heterogeneity. However, their effects can vary between species and they are often neglected in phylogenetic analyses. In addition, a practical problem in the reconstruction of shallow phylogenies is to determine the most efficient set of DNA markers for a reliable estimation. To address these questions, we conducted a multilocus simulation study using empirical values of nucleotide diversity and substitution rates obtained from a wide range of mammals and evaluated the performance of both gene tree and species tree approaches to recover the known speciation times and topological relationships. We first show that deep coalescence can be a serious problem, more than usually assumed, for the estimation of speciation times in mammals using traditional gene trees. Furthermore, we tested the performance of different sets of DNA markers in the determination of species trees using a coalescent approach. Although the best estimates of speciation times were obtained, as expected, with the use of an increasing number of nuclear loci, our results show that similar estimations can be obtained with a much lower number of genes and the incorporation of a mitochondrial marker, with its high information content. Thus, the use of the combined information of both nuclear and mitochondrial markers in a species tree framework is the most efficient option to estimate recent speciation times and, consequently, the underlying species tree.

## Introduction

Resolution of the phylogenetic relationships among organisms, at or above the species level, has usually been performed using mitochondrial DNA (mtDNA) genes [1]. There are several reasons to justify that mtDNA is the most commonly used marker in animal phylogenetics including its elevated substitution rate, which ensures a strong phylogenetic signal, and its reduced effective population size, *N*_e_, which increases the probability of reciprocal monophyly of the gene among different species. In spite of the advantages of mtDNA, the use of a single marker in phylogenetics has a critical limitation: it allows obtaining only one particular genealogy among all possible ones compatible with the true populations or species tree [2], [3]. Thus, making inferences from a single gene could lead, in particular instances, to erroneous biological conclusions.

Indeed, different unlinked loci can have conflicting genealogical histories as a result of the stochastic process of coalescence of alleles due to random genetic drift and incomplete lineage sorting [4], [5]. The probability of observing discordant gene trees depends on *N*_e_ and the branch lengths of the species tree. When these branch lengths (measured in generations) are of the same magnitude as *N*_e_, a situation typically observed in phylogenies of closely related species, deep coalescences in ancestral populations become highly probable and, consequently, the gene tree topology may differ from that of the species tree [6], [7], [8]. The discordance among gene trees can occur not only in topology but also in branch lengths [9]. Noticeably, this source of heterogeneity is always present since different genes are likely to coalesce at different times. This will produce different gene trees (with different branch lengths) even if the topologies are identical. In fact, branch lengths and tree topology heterogeneity can be considered two sides of the same coin and conclusions based on the former can also be applied to the latter. In the last few years, phylogenetics focused on the species tree, rather than on individual gene trees, has received considerable attention since these new methods take into account the coalescence of genes to construct species trees and to estimate reliable split times and demographic parameters [2], [3], [9], [10], [11].

The mutational process is also an important source of variation among gene trees and can significantly affect both classical phylogenetic and species tree reconstruction processes. Short genes and genes with low substitution rates are highly affected by this randomness due to their low information content. Actually, in animals, most nuclear genes have very low substitution rates compared to mitochondrial genes. In this sense, we may raise the question of whether the incorporation of a highly informative marker (e.g., a mitochondrial gene) into a species tree inference can improve the efficiency of the estimates. That is, the addition of this marker may help to reduce the quantity of data (i.e., the number of independent markers) necessary to achieve an adequate level of accuracy in estimating species trees. On the other hand, the incorporation of a mitochondrial gene into the inference might compromise the performance of these methods due to the introduction of a large variance in the substitution rates among loci and a more complex mutational model. It is thus important to determine which properties of this marker predominate in the construction of species trees.

Here, we analyze the influence of deep coalescence and mutational processes in the estimation of divergence times under conditions likely to be encountered in natural populations, mainly focusing on recent mammalian species diversification. For this purpose, we conducted a multilocus simulation study using empirical values of nucleotide diversity and substitution rates obtained from a wide range of mammals. In addition, we examined, under different conditions, the performance of different multilocus strategies (with or without mitochondrial genes) in estimating divergence times in a species tree framework. Finally, we also investigated the power of the different multilocus sets in estimating the species tree topology in the particular situation of a species radiation. The results of these analyses can be useful to make informed choices about the optimal number and types of markers necessary to obtain reliable estimates of species tree parameters in empirical studies of mammals and other groups with similar population sizes.

## Materials and Methods

### General strategy

In order to assess the importance of genealogical and mutational heterogeneity in empirical mammalian phylogenetics, we performed a computer simulation study in the parameter space of typical mammalian diversity. The pipeline designed for the study i) simulates random coalescent gene lineages along the branches of predefined species trees, ii) simulates nucleotide sequence evolution of several loci along the branches of these gene trees, iii) estimates gene trees from concatenated DNA sequence data by a traditional ML phylogenetic method, iv) estimates species tree parameters directly from the DNA sequence data by Bayesian methods that incorporate the process of genealogical sorting, v) calculates both the time to the most recent common ancestor (*t*_MRCA_) of the sampled genes and the divergence times between two species using the data obtained in steps iii and iv, respectively, and vi) assesses the discordance of the estimated times or tree topologies with respect to the corresponding known species phylogeny.

### Gene trees simulation

**Figure 1:**
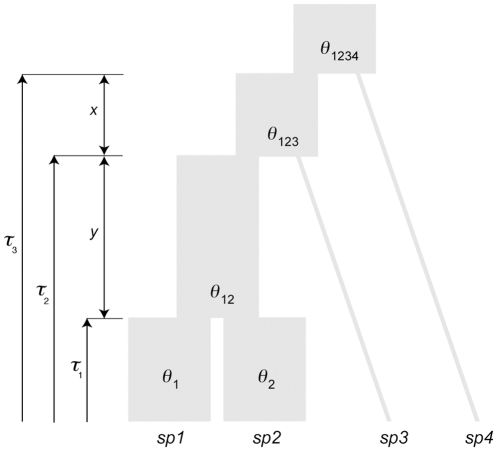
Scheme of the species tree used for computer simulations. *θ*, present and ancestral population size parameters (here, assumed to be constant). *τ*, divergence times. *x* and *y*, length of internal branches. The MCcoal notation of this species tree corresponds to: “(((*sp1*_# *θ*_1_, *sp2*_# *θ*_2_): *τ*_1_ # *θ*_12_, *sp3*): *τ*_2_ # *θ*_123_, *sp4*): *τ*_3_ # *θ*_1234_;”. Both *τ* and *θ* are measured in substitutions/site.

For the main part of this study, we used a 4-taxon, asymmetric bifurcating species tree with no migration (complete isolation model; Figure 1). Gene coalescence was simulated along the branches of this species tree under the neutral coalescence model implemented in MCcoal version 1.2 [12]. The model assumes no recombination within loci and free recombination between nuclear loci. We defined the locus-specific population size parameter, *θ*_g_, as two times the product of the effective number of genes, *N_g_*, and the mutation rate per site per generation, *μ_g_*, in this locus (*θ*_g_ = 2*N_g_μ_g_*). Thus, *θ_nuc_* = *4N_e_μ_nuc_* is the population size parameter of an autosomal loci in a diploid population. For mtDNA genes, due to their clonal and maternal inheritance, this parameter was *θ_mit_* = *N_e_μ_mit_*. Initially, we simulated mitochondrial gene trees under two different *θ_mit_* values, 0.025 and 0.01 (in each case, assuming the same *θ* for all populations of the species tree, i.e. constant *N*_e_). These values encompass a range of typical mammalian values as obtained from the average cytochrome *b* nucleotide diversity in substitutions per site (π_cyt*b*_) estimated in different sources [13], [14], [15]. π_cyt*b*_ = 0.025 corresponds to the average cytochrome *b* nucleotide diversity in surveyed species and π_cyt*b*_ = 0.01 is closer to the median value of the distribution and to the average after excluding rodents, which have the highest diversity values [15]. Under the assumption of selective neutrality and mutation-drift equilibrium, π is an unbiased estimator of *θ* [16]. Similarly, we applied two different per lineage mtDNA substitution rates (*υ_mit_*), 0.01 and 0.02 (in substitutions/site/Myr), which include typical mammalian mitochondrial substitution rates [17]. *υ_mit_* = 0.01 is closer to the average absolute substitution rate of Carnivora, Primates, Insectivora, Cetartiodactyla and Lagomorpha, while *υ_mit_* = 0.02 is more commonly found in Rodentia and Chiroptera [17]. Likewise, we simulated nuclear (autosomal) gene trees with an average substitution rate (*υ_nuc_*) tenfold lower than the mitochondrial value, which is the approximate overall value obtained from empirical data [18]. This gave rise to corresponding *θ_nuc_* values of 0.01 and 0.004.

Because we were interested in estimating only the first split time, *τ*_1_, which is the divergence time between species 1 (*sp1*) and species 2 (*sp2*) (Figure 1), we generated data sets of gene trees with a sample size of 5 sequences per locus for these two species and 1 sequence for the two outgroup species. *τ*_1_ was simulated in 5 time depths: 0.5, 1, 3, 5 and 10 Myr (converted to substitutions/site using the corresponding *υ*). These time depths cover a wide range of both nucleotide substitutions and discordance affecting *sp1* and *sp2*. Two additional species, *sp3* and *sp4*, were used as outgroups. Both internal branches, *x* and *y*, were set as 4 *N_e_* and 8 *N_e_* in number of generations (converted to substitutions/site using the corresponding *μ*), respectively, large enough to minimize incomplete lineage sorting effects in the rooting nodes and therefore to avoid problems with the estimation of the topology of this species tree. For each time depth, 50 gene trees sets were generated for the different markers.

For the analysis of the topology of a rapid species radiation we used the 5-taxon, asymmetric species tree of Huang and Knowles [19]. This is the same tree of Figure 1 with an additional external outgroup species, *sp5*. Internal branch lengths were set to hold two different radiation scenarios, one with very low probability (*x* = 0.5 *N_e_* and *y* = 1.6 *N_e_*), and the other with high probability (*x* = 0.1 *N_e_* and *y* = 0.2 *N_e_*), of obtaining Anomalous Gene Trees [20]. These trees correspond to a moderate and a extreme species radiation, respectively. The split time of *sp1* and *sp2* was set to 8 *N_e_*. The internal branch to *sp5* was set to 12 *N_e_*. Five sequences per locus were simulated for all species except for *sp5*, where only 1 sequence was simulated. We simulated 100 gene trees sets for each of the competing multilocus strategies under the two radiation conditions.

### Multiple sequence alignment simulation

DNA sequence alignments of 1000 base pairs were generated by simulating molecular evolution along the branches of the coalescent gene trees using the Seq-Gen program version 1.3.2. This sequence length was selected as it is close to the length usually sequenced for a typical mitochondrial gene. Sequences were obtained under the HKY85 model of nucleotide substitution [21] with a transition-to-transversion rate ratio, κ [κ = α/β in 21] of 2 and 10 for nuclear and mtDNA loci, respectively [21], [22], and a Gamma distribution with 4 rate categories and a shape parameter of α = 1. The 4 nucleotides were assumed with equal probability. This model was used because it is often observed in real sequences of closely related species.

### Gene trees estimation

ML gene trees were estimated from simulated DNA sequences using RAxML version 7.0.4 [23]. Phylogenetic analyses were conducted specifying the GTRGAMMA model with base frequencies estimated by the program. In the case of multiple loci, the sequences of each independently simulated locus were concatenated and the resulting alignment was analyzed using different substitution models for each partition, as implemented in RAxML [23]. To correct for stochastic substitution rate differences, we made ultrametric trees using the Langley-Fitch method implemented in the program r8s version 1.71 [24] under the assumption of a molecular clock. We then scaled the resulting tree with Ktreedist version 1.0 [25] to have equivalent substitution rates to the initial RAxML tree.

### Species tree estimation

The posterior distributions of the different values of *τ* and *θ* were calculated using the Multilocus Bayesian (MB) Markov chain Monte Carlo (MCMC) method of species tree parameter estimation based on the algorithm of Rannala and Yang [12] and implemented in the program MCMCcoal version 1.2. Since this program does not estimate topologies, the topology of the species tree (Figure 1) was given to the program. Substitution rates and heredity multipliers (which reflect differences in population sizes among loci) were fixed on true values or treated as unknown parameters. The shape and scale parameters of the Gamma priors for *θ* values were set to obtain reasonable confidence intervals (C.I.) reflecting mammalian genetic diversity (mean *θ* = 0.025, C.I._95%_ = 0.003–0.07). Priors for divergence times were set as exponential distributions with the mean equal to the average minimum divergences between *sp1* and *sp2* sequences, estimated from gene trees (see above). In simulations with mitochondrial data, these divergences were obtained only from the mitochondrial tree and further adjusted to the nuclear mean with the corresponding *υ* ratio between these two markers. We ran the MCMC analyses for 1,000,000 generations, sampling every 100 generations, with 150,000 generations of burn-in. To achieve reasonable acceptance proportions in all experimental conditions, we adjusted the fine-tune parameters for proposals in the MCMCcoal control file when necessary (as specified in the MCMCcoal documentation).

Finally, we estimated the species tree topology from the alignments simulated under the rapid radiation scenario using *BEAST software [26]. We ran the MCMC analyses for 30,000,000 and 20,000,000 generations for the samples with and without a mitochondrial marker, respectively, sampling every 1000 generations, and with 5% generations of burn-in. These conditions ensured that convergence was achieved in all simulations. We then calculated the mean Robison-Foulds (RF) distance (that is, the number of different partitions) between the estimated topology and the true (simulated) species tree and the proportion of correct topologies in each simulation set.

### Analysis of simulated data

As a summary parameter of the amount of gene tree and species tree discordance we used the parameter *δ*, which we defined as the difference (in percent) between the *t*_MRCA_ of the *sp1* and *sp2* sampled genes (obtained from ML gene trees) and the true speciation time. Since errors will be larger for older divergences, the percent error with respect to the true split time provides a more realistic estimate of the performance of the different conditions at different divergences than the simple difference between split times. Using the average value of the *δ* parameter, estimated from the 50 replicates, we quantified the effect of gene tree coalescence and mutational processes and measured the maximum percent error in split time estimation expected in phylogenetic methods.

To evaluate the performance of the MB method we calculated, for each simulation setting and time depth, both the average percent error of the 50 divergence time estimates and the lowest and the highest percent error still within the range defined by the lower and upper inter-quartiles (IQ)±1.5 times the inter-quartile range (IQR). We notice that these MB estimates cannot be directly compared with the parameter *δ*. The possibility of taking into account high levels of gene tree discordance by considering gene genealogies in the Bayesian inference allows us to obtain unbiased estimates of the species divergence time whereas *δ* estimated from the gene trees symbolizes the average of the maximum expected error in the phylogenetic approach and it will be, therefore, always older than the true species split time.

## Results

### Impact of deep coalescence on the estimation of speciation times given actual mammalian diversity parameters

**Figure 2:**
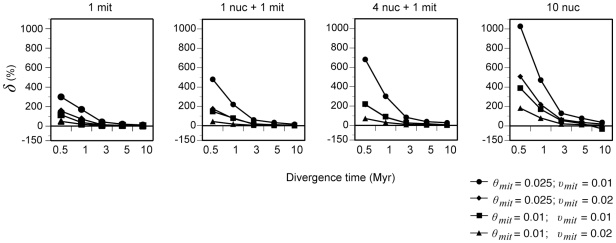
Expected levels of gene tree discordance under different multilocus strategies and different combinations of mammalian evolutionary parameters. *δ*, % difference between the *t*_MRCA_ of the sampled genes and the species split time. mit, mitochondrial. nuc, nuclear. *θ_mit_*, population size parameter for mitochondrial genes. *υ_mit_*, substitution rate for mitochondrial genes. The corresponding population size parameter for nuclear genes was 2.5 times smaller. The nuclear substitution rate was tenfold lower in each case.

In order to estimate the levels of deep coalescence expected in shallow phylogenies of mammals, we simulated genealogies and nucleotide sequence alignments for four sets of multiple loci and at different divergence times, using average diversity parameters obtained from a wide range of mammalian species [13], [14], [15]. We then estimated split times from gene trees as the *t*_MRCA_ of the *sp1* and *sp2* genes and compared them with the known speciation time (Figure 1). The differences found must be due to the combined effects of both coalescence and nucleotide substitution saturation although, at these genetic distances, most of the differences should be attributed to coalescence. Results clearly demonstrate, as shown by the *δ* parameter, that gene coalescence is a serious problem in the estimation of divergence times for mammalian species and, under certain conditions typically found in mammals, the levels of overestimation can be much higher than usually assumed (Figure 2). For example, in conditions with the highest population sizes (*θ_mit_* = 0.025 and *υ_mit_* = 0.01) the measured mtDNA divergence is 2.7 Myr on average for a simulated species split of 1 Myr, meaning that there is an amount of discordance (*δ*) in the estimated time of 170%. For the shallowest divergences (0.5 Myr), *δ* reaches 300% for a single mitochondrial gene. These values are in agreement with those predicted by coalescence theory (discordance should be between *N_g_* and 2*N_g_* depending on the specific genealogy), indicating that genealogical discordance is indeed the major cause of these errors. In addition, the introduction of additional nuclear markers in the classical phylogenetic inference yielded increasing overestimates of divergence times due to the higher coalescence times of the nuclear genes, with the worst estimations being obtained with the strategies that only used nuclear genes (Figure 2). Overall, although coalescence is not a problem for mammalian divergences ≥10 Myr, *δ* is quite high in most situations for divergences ≤3 Myr, a timeframe which encompasses many typical mammalian speciation times [27]. Thus, classical phylogenetic methods can lead to serious errors in the estimation of species split times of closely related mammalian species for all multilocus strategies.

### Multilocus coalescent approach: number and types of mammalian markers necessary to estimate speciation times

**Figure 3:**
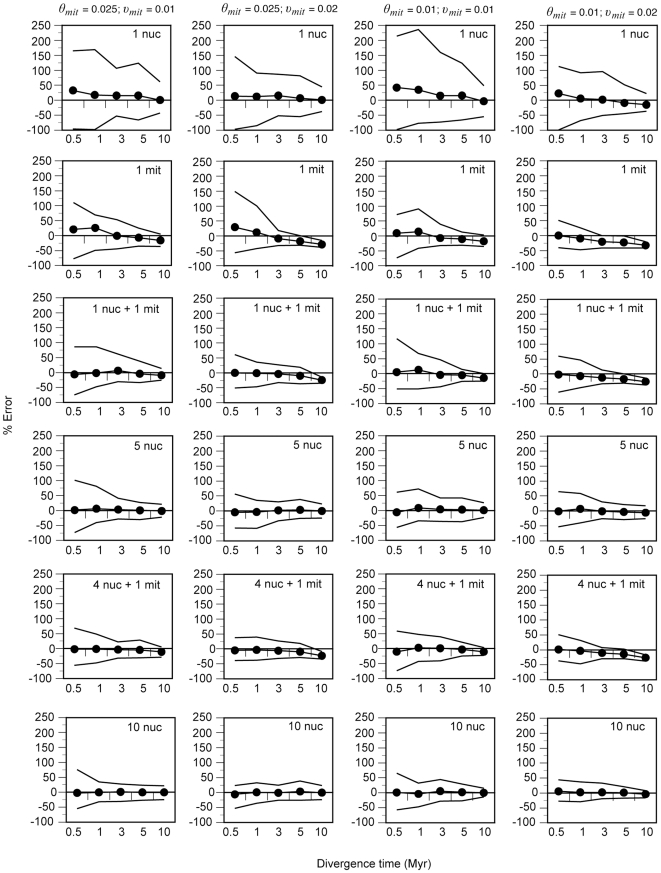
Performance of the Bayesian (MB) approach under different multilocus strategies and different combinations of mammalian evolutionary parameters. Sample size, n = 5. The line with circles shows the average percent error among the 50 replicates. Solid lines indicate the lowest and the highest estimate still within the range IQ±1.5 IQR. Columns show the different diversity parameter combinations and rows correspond to the different multilocus strategies. mit, mitochondrial. nuc, nuclear. *θ_mit_*, population size parameter for mitochondrial genes. *υ_mit_*, substitution rate for mitochondrial genes. The corresponding *θ* for nuclear genes was 2.5 times smaller. The nuclear substitution rate was tenfold lower in each case.

Given the previous results, we used a multilocus, coalescent-based, Bayesian method of species tree reconstruction to assess the question of the optimal number and types of mammalian markers necessary to obtain reliable estimates of the species tree (divergence time of *sp1* and *sp2*). For this purpose, we first investigated the performance of several representative multilocus strategies in order to choose the best number and combination of markers (Figure 3). Our results show that the MB approach provides accurate estimations of speciation times given typical mammalian conditions, and much better than the classical phylogenetic methods. The variance of these estimates (as measured by the range defined by the lowest and the highest estimate still within the range IQ±1.5 IQR) can be quite large but the differences among experiments show very informative patterns. Thus, the variance decreases from recent to more distant divergences in all simulations due to the relatively smaller effect of coalescence in the latter. As expected, the variance also becomes lower in conditions with smaller effective population sizes. In addition, the higher precision obtained in conditions with high substitution rates (*υ_mit_* = 0.02) is likely due to the presence of more informative sites in the alignments. Finally, there is an important decline in the variance with the incorporation of additional nuclear loci. Thus, the use of multiple nuclear loci in the inference has the opposite consequence than in the case of the phylogenetic approach: it significantly improves the performance in very recent divergences.

Interestingly, although the strategy of sampling 10 nuclear genes is the best option among the multilocus strategies analyzed here, we obtained very similar results with 4 nuclear loci and 1 mtDNA marker. In contrast, the other strategy with 5 markers (5 nuclear loci) yielded slightly more variance in the estimates than the mixed strategy, particularly in the shallowest divergences. The potential problems of mtDNA (such as a slight saturation observed in the oldest divergences, which manifests as an underestimation of divergence times) are clearly outweighed by its advantages, pointing to the utility of a mitochondrial gene in improving the resolution of mammalian shallow phylogenies with less empirical effort. Therefore, we compared the performance of the two best multilocus strategies, i.e., a large number of nuclear genes (10 nuclear genes) and a mixed strategy (4 nuclear genes and 1 mitochondrial gene), in estimating divergence times. For this purpose, we evaluated these two strategies in the presence of high levels of gene tree discordance (*θ_mit_* = 0.025 and *υ_mit_* = 0.01) and using conditions typically found in different empirical situations.

### Sample size

**Figure 4:**
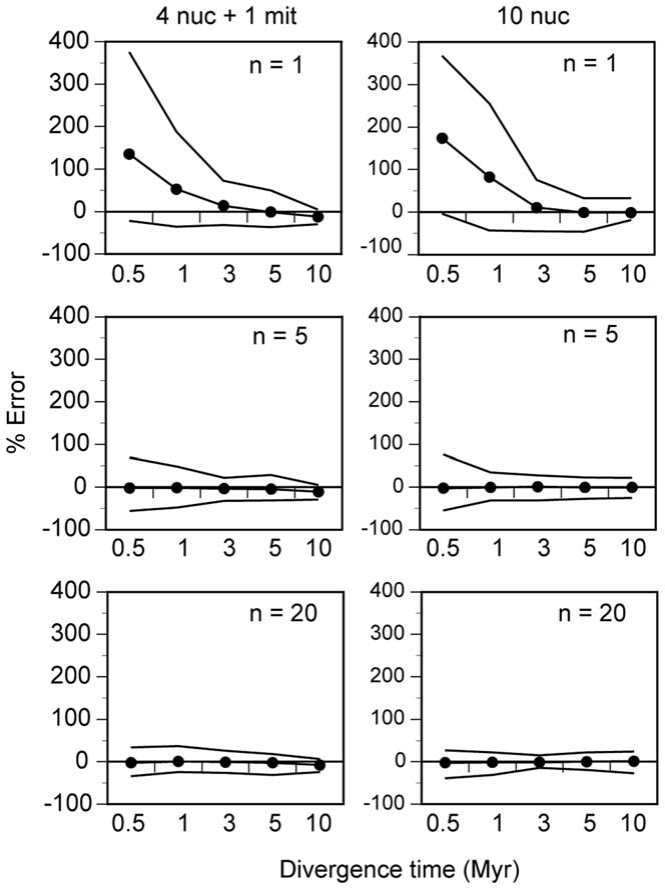
Performance of the Bayesian (MB) approach under different sample sizes. *θ_mit_* = 0.025. *υ_mit_* = 0.01. The population size parameter for nuclear genes was 2.5 times smaller. The nuclear substitution rate was tenfold lower. The line with circles shows the average percent error across replicates. Solid lines indicate the lowest and the highest estimate still within the range IQ±1.5 IQR. n, number of sequences sampled per locus.

To examine the effect of sample size in the performance of the MB method with and without mtDNA information, we simulated two additional sets with 1 and 20 sequences per nuclear locus, respectively, and compared the results with the simulation set of 5 sequences per nuclear locus. The results indicate that the addition of more sequences equally improves the estimation and the variance of the divergence times in the two multilocus strategies (Figure 4). In addition, samples with 20 sequences per nuclear locus improve the performance of both strategies but in a much smaller magnitude than in the case of increasing from 1 to 5 sequences per nuclear locus. This effect is especially marked for recent divergences. Clearly, the strategy of employing only one sample per nuclear locus is a very unsatisfactory option. Thus, the number of loci necessary to obtain reliable estimates from 1 sequence per locus must be considerably higher than 10, too far from typical samples in empirical mammalian phylogenetics. In summary, both the 10 nuclear loci and the 5 mixed loci strategies continue to perform similarly with different sequences per nuclear locus.

### Among-loci rate variation

In the previous sections, we assumed that the differences in substitution rates among loci (in our case, the difference between mitochondrial and nuclear rates) were known and, therefore, we set the true values as fixed in the process. Here, to be as close as possible to a real empirical situation, we treated the substitution rate of the different markers as unknown parameters. We then estimated the unknown rates in two different ways: i) the rates were internally estimated by the MB method (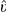
MB), and ii) the ratios of rates were externally calculated from an ML tree (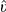
ML) and then fixed in the MB method. In addition, we simulated nuclear markers with two different substitution rates: *υ_nuc_* = 0.001 (the rate used in previous simulations) and *υ_nuc_* = 0.0005, as representatives of different types of nuclear markers such as introns and exons, respectively [18]. Furthermore, *υ* was allowed to vary randomly among nuclear loci ranging from 0.7 to 1.4 times around the corresponding mean as it happens with typical markers [18]. Finally, we also treated the heredity multiplier as an unknown parameter since in natural conditions many factors might cause that this parameter deviates from the expected value [28].

**Figure 5:**
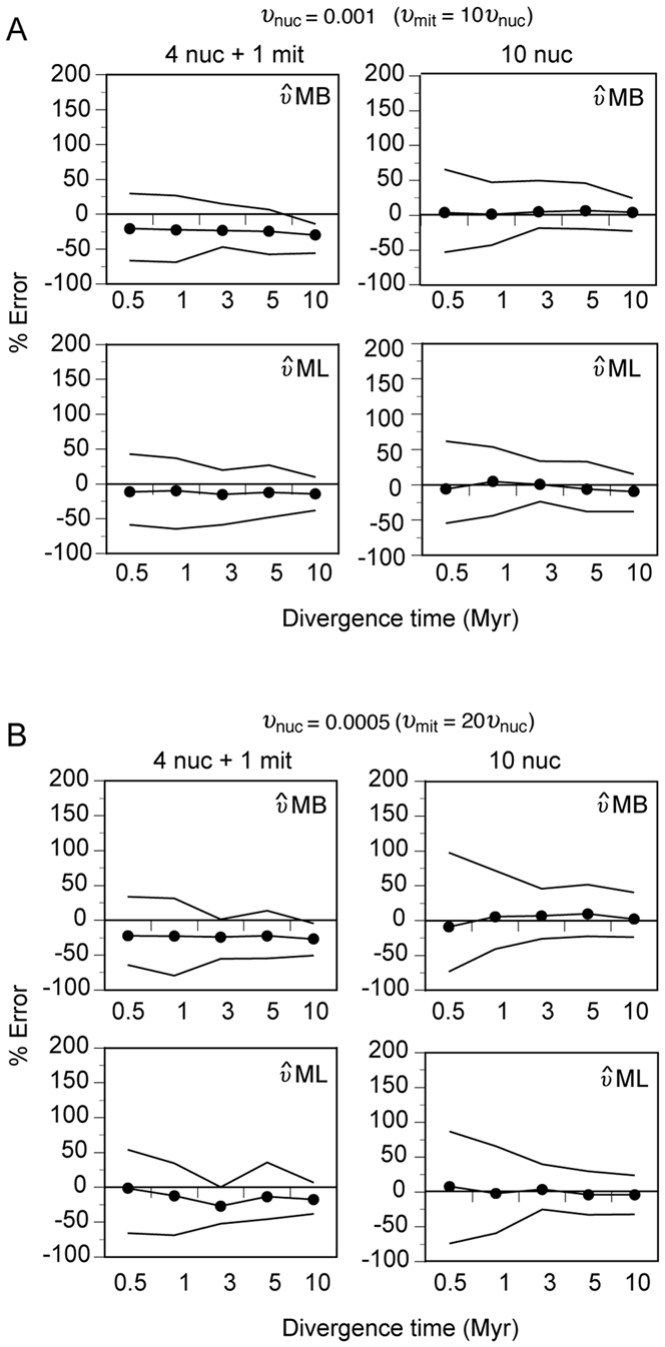
Performance of the Bayesian (MB) approach without fixing mutation rates and heredity multipliers. *θ_mit_* = 0.025. *υ_mit_* = 0.01. The population size parameter for nuclear genes was 2.5 times smaller. Nuclear markers were simulated with two different average substitution rates: *υ*_nuc_ = 0.001 (A) and *υ*_nuc_ = 0.0005 (B). n = 5. The line with circles shows the average percent error across replicates. Solid lines indicate the lowest and the highest estimate still within the range IQ±1.5 IQR. 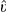
MB, substitution rates estimated by the Multilocus Bayesian (MB) software. 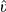
ML, substitution rates estimated from Maximum Likelihood (ML) individual gene trees.

Figure 5 shows the results for the different conditions and the two competing multilocus strategies examined. In all conditions, the MB method with 10 nuclear genes was able to accurately estimate divergence times in spite of the among-nuclear rate variation, although the precision was not as good as in the case of an equal and known rate (Figure 5A and B; 10 nuc, 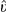
MB). As expected, the precision of the estimates is notably lower in the case of *υ_nuc_* = 0.0005, a condition that would be typical for highly conserved introns or nuclear exons. The calculations of split times do not greatly improve if the rates are estimated from the ML trees (Figure 5A and B; 10 nuc, 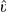
ML). On the other, estimates from the hybrid marker strategy are systematically biased (Figure 5A and B; 4nuc+1 mit, 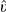
MB), likely because both the prior for *υ* and the simple substitution model implemented in MCMCcoal cannot take into account the large differences between the mitochondrial and the nuclear substitution rates, producing a systematic underestimation of the divergence times. However, our results show that this bias can be partially attenuated by calculating the substitution rates from the ML gene trees, which raises the levels of accuracy closer to those obtained with the multilocus nuclear approach (Figure 5A and B; 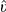
ML). Thus, in recent divergences we can obtain good estimates of the split times using the hybrid approach and with less variance than in the case without mitochondrial information by calculating the rates externally. The improvement in variance is particularly noteworthy when using exon-like nuclear markers (*υ_nuc_* = 0.0005), helping to overcome the low information content of these markers. Thus, in conditions with little or no information about critical parameters, which could be the most frequent ones in real data sets, the precision of the estimates can be increased without obtaining highly biased values by using mitochondrial data in an appropriate manner.

### Topology estimation of species radiations

Finally, we also compared the performance of the two competing multilocus strategies in estimating the topology of species trees in a rapid radiation [19]. Table 1 shows the results of the *BEAST analyses for two different diversification scenarios. We selected two difficult radiations to highlight differences among both strategies and therefore the errors in estimating the correct topology are high for all cases studied. However, in the moderate radiation both data sets provided a similar performance in estimating the species tree topology, as in the case of estimating divergence times. On the other hand, the fast radiation condition already shows differences among both data sets, with the multinuclear strategy with 10 markers yielding a slightly higher percentage of correct species tree topologies. A similar result was observed with RF distances to the real tree. These results suggest that, to resolve rapid radiations, the best approach should be sampling an increasing number of nuclear markers.

**Table 1:** Results of the *BEAST analysis with different multilocus strategies and species radiations.

Multilocus strategy	Radiation	% correct topologies	RF
4 nuclear+1 mitochondrial	Moderate	65	0.7
4 nuclear+1 mitochondrial	Fast	10	2.74
10 nuclear	Moderate	67	0.68
10 nuclear	Fast	17	2.28

The branch lengths of the trees corresponding to the moderate and fast radiations are given in Materials and Methods.

## Discussion

### Gene tree heterogeneity as the null hypothesis in the inference of shallow mammalian phylogenies

One of the questions we have addressed in this work is the specific impact of gene tree discordance in empirical phylogenetic studies of mammals. Our results indicate that, even in the absence of gene flow and other factors generating gene tree heterogeneity, the estimation of speciation times in recently diverged species can be greatly affected by genealogical discordance. This effect is obviously more pronounced for nuclear genes due to their higher population sizes but, even for mitochondrial genes, commonly used to date recent divergences, the split times estimated with classical gene tree approaches can be severely biased. Thus, with typical mitochondrial nucleotide diversity values observed in mammals (the mean per site cytochrome *b* nucleotide diversity obtained from 138 mammalian species is π_cyt*b*_ = 0.025 [15]), the overestimation of recent divergence times with phylogenetic approaches is much higher than usually assumed (Figure 2). This means, for instance, that a Pleistocene split time can be estimated as a much older one, e.g. in the Pliocene. This change of geological epoch in the estimation of speciation times may have important consequences to understand the factors that promote speciation [27]. In the case of the shallowest divergences that we simulated (0.5 Myr), the amount of discordance can reach even 300% for mitochondrial genes (that is, a split time can be erroneously estimated as four times higher). At these short divergences the estimated times should be exclusively treated as times to the most recent common ancestor of the sequences and they should not be used as speciation or population split times.

It should be remarked that, given typical population sizes in mammals, these problems are most serious below 3 Myr and they almost disappear by 10 Myr (Figure 2). Thus, it is difficult that gene tree discordance would be a problem to date split times of deep mammalian lineages such as those affecting different orders or, at least, it should be a much smaller problem than other phylogenetic artifacts such as long branch attraction [29]. However, further studies may be necessary to specifically address the effect of incomplete lineage sorting on nuclear gene trees at deeper nodes than the ones studied here [30].

Another interesting point is that it has been observed in some cases that dates obtained with mitochondrial genes are older than those obtained with nuclear genes [31], in contradiction with the expected higher coalescence times of nuclear genes. It seems that, when dating is performed with mitochondrial genes and calibration points are placed in deep nodes of the tree, the likely saturation of mitochondrial sequences in the deepest branches may have the effect of artifactually lowering substitution rates of the whole tree and thus of increasing the dates of shallow nodes. If this effect is greater than coalescence, then mitochondrial datings can be older than nuclear datings. Therefore, the choice of calibration points is also important for dating recent divergences.

The errors in estimating split times due to lack of recognition of gene coalescence will vary, of course, depending on the relation between the split time of the species and their effective population size. For example, in some mammalian orders such as Rodentia (π_cyt*b*_ = 0.033), Lagomorpha (π_cyt*b*_ = 0.023) and Chiroptera (π_cyt*b*_ = 0.020), the high values of mitochondrial nucleotide diversity in some species [15] may lead to extremely elevated expected population sizes. Species in these groups are, indeed, among those with the largest populations of all mammals and therefore they are prone to high incongruences in gene trees. However, other demographic factors, such as bottlenecks, can also raise the levels of incongruence in gene trees, making necessary to consider the importance of coalescence even in the cases of species with apparently small populations.

Our results also indicate that, in the phylogenetic approach, increasing the number of nuclear genes does not improve the inference of split times and that the combination of mitochondrial and nuclear markers can be even worse than using only one mitochondrial locus, as expected (Figure 2). The best solution is, therefore, the use of species tree approaches [2], [3], [9], [10], [11]. As clearly indicated in our analyses, failure to do so may lead to large overestimations of recent mammalian speciation times.

### Mammalian mtDNA as an additional maker in the multilocus coalescent inference

Given that phylogenetic studies of closely related species should be performed using multilocus species tree approaches, we next searched for the best combination of markers to obtain accurate estimations of split times and topological relationships with these methods. Specifically, the incorporation of a mitochondrial gene such as cytochrome *b* in nuclear multilocus strategies may have advantages due to its high information content, but also some disadvantages due to the heterogeneity of evolutionary modes introduced. Regarding the latter aspect, one could think that using a homogenous data set with similar evolutionary rates and models (i.e. all nuclear genes) should perform better. Thus, it is necessary to test the performance of species tree methods upon the addition of mitochondrial genes and see which properties of this marker predominate. Our analyses show that the more complex substitution pattern of mtDNA does not greatly influence parameter estimation. That is, saturation is not important in the range of divergence times where deep coalescence is important. However, the extremely different evolutionary rates between mtDNA and nuclear loci in mammals could be a greater problem. In this respect, we have also shown that the calculation of relative evolutionary rates from external gene trees, either to set these estimates as fixed values or to be used as more informative priors, can greatly help in the estimations of split times. Thus, the high information content of mtDNA is highly advantageous in speciation time estimation and clearly outweighs its potential problems if properly addressed.

However, there are also situations where the use of the highest possible number of unlinked loci outperforms the set of a reduced number of nuclear genes with an additional mitochondrial marker. This is the case, as we have shown, in the estimation of tree topologies of rapid radiations. It has been known for long that a high number of independent loci is necessary to resolve difficult trichotomies [32]. We show here that this is especially true for rapid radiations and that the addition of a variable mitochondrial gene is not as helpful as in the estimation of split times. In addition, it should be noted that some trichotomies with very short divergence times among splits might not be possible to resolve even with genomic data. Although the exact results we obtained with the different multilocus strategies are not necessarily going to hold in all empirical situations, since many other factors not tested here may influence the results, the tendencies observed in our simulations may be useful to guide the sampling design in studies of mammals and other groups with similar population sizes.

In summary, mtDNA can be a very useful marker for population divergence and recent diversification inferences in multilocus approaches. In some situations it might help as just one more marker but, in others, its high information content can improve the estimations of species tree parameters, such as split times, with less sampling effort. It is also clear, as often stated [33], that the use of a single mitochondrial gene is inappropriate for these tasks. However, if we are able to embrace its good properties and to add them to those of the multinuclear coalescent approach, we can improve the reconstruction of the past evolutionary history of numerous species. Further empirical studies are necessary to uncover all the potential of these approaches.
